# Reduced neural feedback signaling despite robust neuron and gamma auditory responses during human sleep

**DOI:** 10.1038/s41593-022-01107-4

**Published:** 2022-07-11

**Authors:** Hanna Hayat, Amit Marmelshtein, Aaron J. Krom, Yaniv Sela, Ariel Tankus, Ido Strauss, Firas Fahoum, Itzhak Fried, Yuval Nir

**Affiliations:** 1 Department of Physiology and Pharmacology, Sackler School of Medicine, Tel Aviv University, Tel Aviv, Israel; 2 Sagol School of Neuroscience, Tel Aviv University, Tel Aviv, Israel; 3 Department of Anesthesiology and Critical Care Medicine, Hadassah-Hebrew University Medical Center, Faculty of Medicine, Hebrew University of Jerusalem, Jerusalem, Israel; 4 Functional Neurosurgery Unit, Tel Aviv Sourasky Medical Center, Tel Aviv, Israel; 5 Sackler Faculty of Medicine, Tel Aviv University, Tel Aviv, Israel; 6 EEG and Epilepsy Unit, Department of Neurology, Tel Aviv Sourasky Medical Center, Tel Aviv, Israel; 7 Department of Neurosurgery, University of California Los Angeles, Los Angeles, CA USA; 8 Department of Biomedical Engineering, Faculty of Engineering, Tel Aviv University, Tel Aviv, Israel; 9 The Sieratzki-Sagol Center for Sleep Medicine, Tel-Aviv Sourasky Medical Center, Tel-Aviv, Israel

**Keywords:** Sleep, Sensory processing

## Abstract

During sleep, sensory stimuli rarely trigger a behavioral response or conscious perception. However, it remains unclear whether sleep inhibits specific aspects of sensory processing, such as feedforward or feedback signaling. Here, we presented auditory stimuli (for example, click-trains, words, music) during wakefulness and sleep in patients with epilepsy, while recording neuronal spiking, microwire local field potentials, intracranial electroencephalogram and polysomnography. Auditory stimuli induced robust and selective spiking and high-gamma (80–200 Hz) power responses across the lateral temporal lobe during both non-rapid eye movement (NREM) and rapid eye movement (REM) sleep. Sleep only moderately attenuated response magnitudes, mainly affecting late responses beyond early auditory cortex and entrainment to rapid click-trains in NREM sleep. By contrast, auditory-induced alpha–beta (10–30 Hz) desynchronization (that is, decreased power), prevalent in wakefulness, was strongly reduced in sleep. Thus, extensive auditory responses persist during sleep whereas alpha–beta power decrease, likely reflecting neural feedback processes, is deficient. More broadly, our findings suggest that feedback signaling is key to conscious sensory processing.

## Abstract (web summary)

Intracortical recordings in humans reveal that auditory stimulation during sleep induces robust spiking and high-gamma responses, whereas alpha–beta desynchronization—likely reflecting neural feedback processes—is reduced compared to wakefulness.

## Main

Sleep is defined as a reversible, homeostatically regulated state of reduced behavioral responsiveness to environmental stimuli^1,2^. A high arousal threshold in response to external sensory stimulation is the main criterion defining sleep, especially in nonmammalian species such as fish or flies where sleep cannot be determined via electroencephalogram (EEG) criteria^3–5^. However, the extent to which sleep affects responses along sensory pathways remains unclear. On one hand, responses to external stimuli in cortical sensory regions may be attenuated during sleep, given that perception of external events is rarely reported upon awakening, and stimuli are not incorporated often in dream content^6^. On the other hand, other lines of evidence suggest robust responses during sleep, since discriminative processing persists for behaviorally relevant or semantic incongruent stimuli^7–15^ as well as for contextual cues in targeted memory reactivation^16,17^. In addition, recent animal studies reporting comparable responses in the primary auditory cortex (A1) to stimuli across sleep and wakefulness have challenged the long-held assumption that natural sleep limits an effective relay to sensory cortex (‘thalamic gating’) as is the case for deep anesthesia^18–25^. Whether this is also the case in consolidated human sleep remains unknown, since it is possible that robust auditory responses reflect a sentinel-like process that is unique to fragmented sleep in prey animals.

Previous studies that attempted to address this question using magnetoencephalography (MEG)^26,27^, EEG^28–30^ and functional magnetic resonance imaging (fMRI)^10,31^ in humans have a number of limitations. Brief stimulation during sleep elicits a large stereotypical response—an evoked slow wave often followed by a sleep spindle, known as a ‘K complex’—that masks the precise dynamics and limits data interpretation. The spatial and temporal resolutions of EEG and fMRI, respectively, cannot distinguish the neuronal sources of early (<150 ms) selective auditory responses from late (~200–1000 ms) nonspecific sleep responses^32^, or determine whether sleep predominantly affects feedforward or feedback processing.

Intracranial recordings in humans could potentially overcome many of these limitations; for example, a recent human study in light anesthesia reported disruption in auditory responses beyond the primary cortex upon loss of consciousness^33^, but whether this is also the case during natural sleep remains unclear. To investigate this and overcome existing limitations, we capitalized on a unique opportunity to compare auditory responses in neurosurgical epilepsy patients implanted with depth electrodes when they were awake or naturally sleeping while we intermittently presented auditory stimuli. Our results establish robust auditory spiking and high-gamma responses during sleep across the temporal lobe and reveal substantial differences in alpha–beta power decreases, which are prevalent in wakefulness but strongly disrupted in sleep.

## Results

**Fig. 1:**
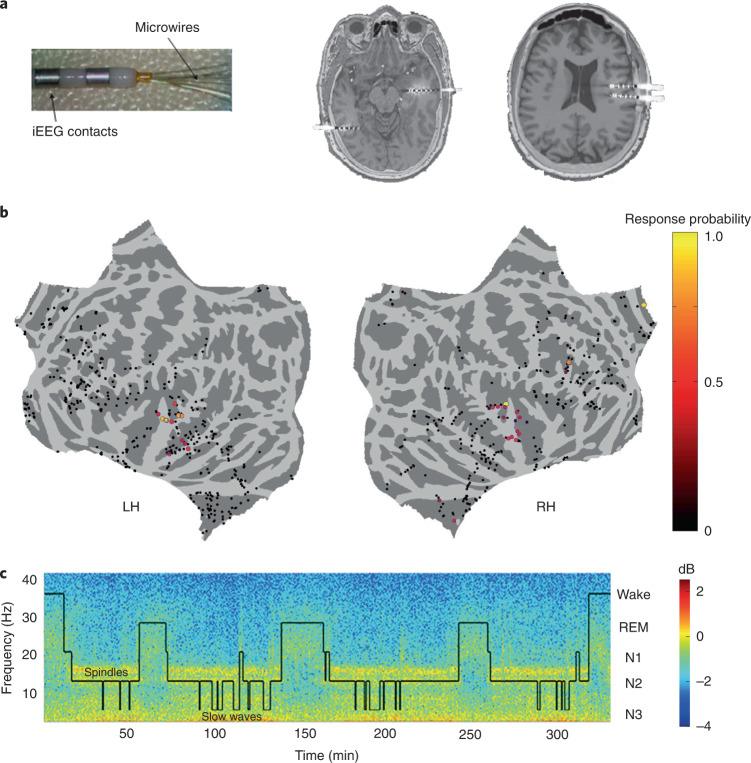
Experimental paradigm. **a**, Left, depth electrodes (6–12 per patient) implanted in patients with epilepsy for clinical monitoring, each consisting of eight 1.5-mm iEEG contacts along the shaft and eight 40-μm microwires protruding from the distal tip, recording LFP and spiking activities. Right, two representative pre-implant magnetic resonance images co-registered with post-implant computed tomography used to localize electrodes from the same individual. **b**, A topographic display (flat cortical map) of all sites where neuronal activity was recorded (each circle denotes one iEEG macroelectrode or a bundle of microwires) along with the probability of observing an auditory response in wakefulness (number of responses/number of stimuli played, color bar on right). LH, Left Hemisphere; RH, Right Hemisphere. **c**, Representative time–frequency representation (spectrogram) of iEEG recorded in one individual during a full-night sleep study with intermittent auditory stimulation. Warm colors (for example, red, see color bar on far right) mark increased power in specific time–frequency windows (frequency shown on left side of *y* axis). Superimposed hypnogram (black trace) marks the time-course of sleep/wake states (shown on right side of *y* axis). Note that NREM stages N2 and N3 are associated with increased power in spindle (10–15 Hz) and slow (<4 Hz) frequency ranges.

**Extended Data Fig. 1:**
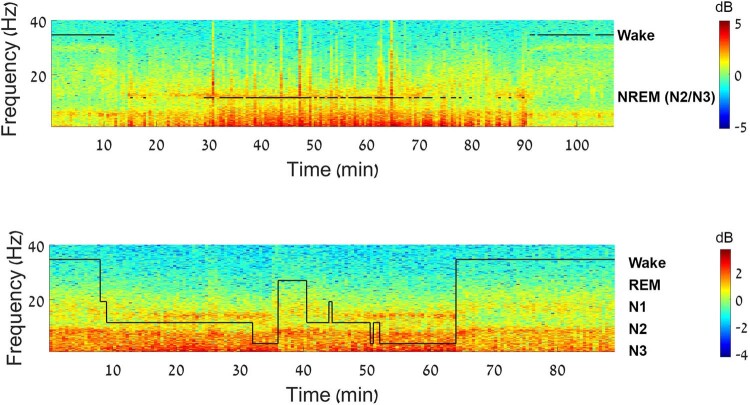
Sleep scoring. Representative time–frequency representation (spectrogram) of iEEG recorded during a nap session. Warm colors (for example red) indicate increased power in specific time–frequency windows (frequency shown on left side of y-axis). Superimposed hypnograms (in black) present the time-course of sleep/wake states (shown on right side of y-axis); top, one nap session with automatic sleep scoring; and bottom, one nap session with full PSG.

To compare auditory responses in wakefulness and natural sleep in humans, we recorded intracranial electroencephalograms (iEEGs, *n* = 987 contacts), microwire local field potentials (LFPs, *n* = 937 microwires) and neuronal spiking activity (*n* = 713 clusters) from multiple cortical regions (Fig. 1a,b) in 13 patients with drug-resistant epilepsy implanted with depth electrodes for clinical monitoring (14 sessions, including 8 full-night sessions lasting 484.8 ± 45.99 min, and 6 daytime nap sessions lasting 103.6 ± 7.7 min). At least one depth electrode in each monitored individual targeted auditory (or other lateral temporal) cortical regions. We intermittently presented auditory stimuli, including clicks, tones, music, words and sentences, via a bedside speaker during the same recording session while participants were awake and asleep (Fig. 1c and Supplementary Table 1). Sound intensity level was adjusted before each session such that stimuli were clearly perceived (well above threshold) yet minimally disruptive, and kept fixed throughout overnight recordings. Sleep/wake stages were scored according to established guidelines^34^ (Fig. 1c and Extended Data Fig. 1) based on full polysomnography (PSG) including electrooculogram, electromyogram, scalp EEG and video monitoring whenever possible (*n* = 7 sessions), as previously described^35^, or EEG/iEEG and video (*n* = 7 sessions; Methods). To distill the changes in auditory responses associated with sleep, rather than the absence of an explicit task or participant report, we employed a passive auditory stimulation paradigm in wakefulness while recording neuronal activity across multiple sites and cortical lobes. In these conditions, auditory responses were predominantly observed in the lateral temporal lobe (Fig. 1b).

### Robust auditory spiking and gamma responses during non-rapid eye movement (NREM) sleep

**Fig. 2:**
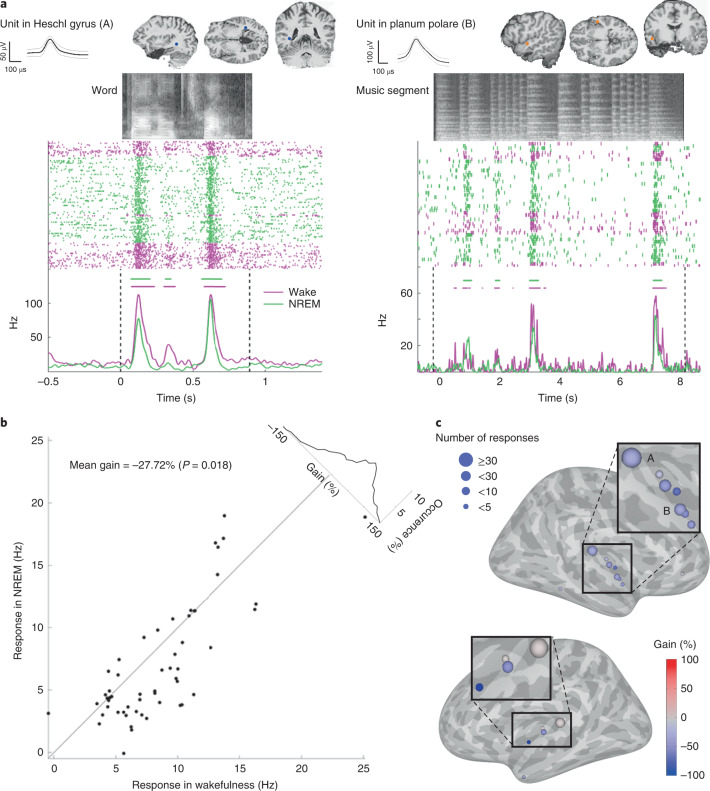
Robust auditory spiking responses across the temporal lobe during NREM sleep. **a**, Left, representative spiking response of neuronal unit in response to word in the primary auditory cortex. The top row shows the action potential waveform (left inset, mean ± s.d.) and the anatomical location of the recorded unit (right inset, circle in MRI sections), while the grayscale soundwave spectrograms are shown above the raster (lighter shades denote stronger power). Pink, wakefulness; green, NREM sleep. Vertical dotted black lines mark stimulus onset and offset. Horizontal bars above peri-stimulus time histogram (PSTH) time-courses indicate automatically detected response intervals for which response magnitude was compared quantitatively. Right, same format for a unit in higher-order auditory cortex (planum polare) responding to music. **b**, Scatter plot of auditory spiking response magnitudes during NREM sleep (*y* axis) versus wakefulness (*x* axis), together with a histogram of gain values comparing response magnitudes (upper-right corner along the diagonal). *N* = 312 responses/55 clusters/7 patients. Each data point represents the averaged response across stimuli and trials per cluster. Mean and *P* value were calculated using a nested mixed model analysis (Methods) (confidence interval (CI) (−43.381, −12.064), *P* = 0.018). **c**, Gain values of spiking response magnitudes (NREM versus wakefulness) in each region exhibiting auditory responses. The position of each circle denotes its anatomical location shown on a standard (Montreal Neurological Institute (MNI)) brain template, the circle’s color represents the average gain detected in that region (color bar on bottom right), and the circle’s size reflects the number of responses detected in that region. The letters A and B mark the locations of the representative units shown in panels **a** and **b**. Source data

**Extended Data Fig. 2:**
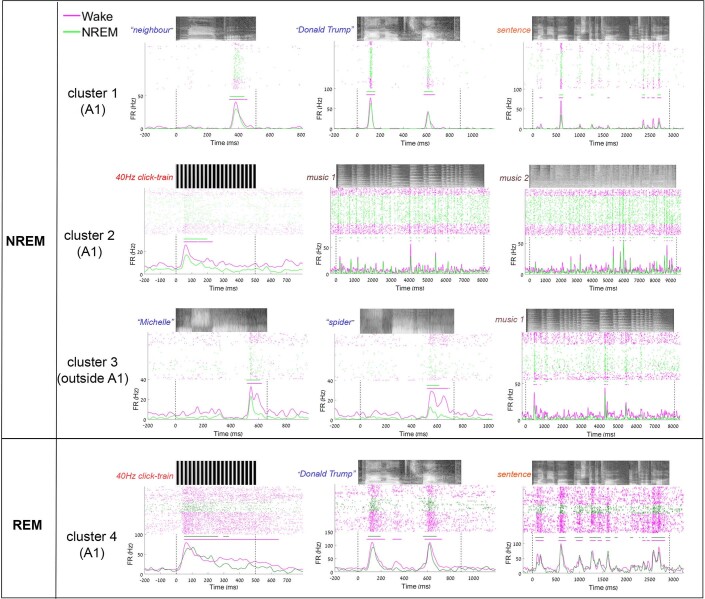
Additional examples of neuronal auditory responses during wakefulness and sleep. Representative raster plots and PSTHs of unit spiking activities in auditory cortex in response to auditory stimuli (words in dark blue, sentence in orange, 40 Hz click-train in red, music in purple) during wakefulness (pink) and NREM sleep (light green) or REM sleep (dark green). Grayscale soundwave spectrograms are shown above each raster (lighter shades denote stronger power). Vertical dotted black lines mark stimulus onset and offset. Horizontal bars above PSTH time-courses indicate automatically-detected response intervals (Methods) for which the response magnitudes were compared quantitatively.

**Extended Data Fig. 3:**
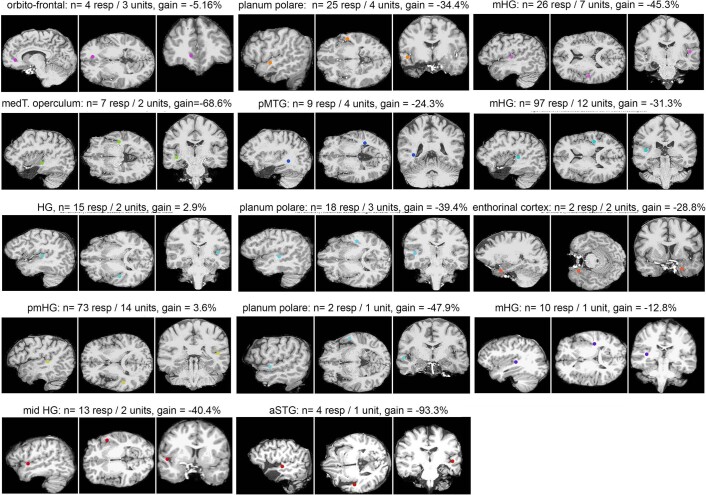
Anatomical location of auditory-responsive units. Each triplet of brain images shows sagittal (left), axial (middle), and coronal (right) MRI sections. Colored dots denote the location of the microwire bundle as identified by co-registration of post-implant CT with pre-implant MRI (Methods), using native (individual) patient coordinates. Abbreviations: mHG=medial Heschl Gyrus; medT operculum=medial Temporal operculum; pMTG= posterior Middle Temporal Gyrus; mid HG = middle Heschl Gyrus; aTG= anterior Superior Temporal Gyrus.

We recorded spiking activity from 713 neuronal clusters, of which 55 clusters (7.7%, from 7 patients) produced a significant auditory response (increased firing rate compared with baseline, *P* < 0.01 by Wilcoxon–Mann–Whitney test) to at least one stimulus in at least one vigilance state (Fig. 2a; see Extended Data Fig. 2 for additional examples). A nested linear mixed model analysis (used as the main statistical approach throughout; Methods) revealed that, on average, the magnitude of spiking responses during NREM sleep was decreased by −27.72% compared with wakefulness (*P* = 0.018; Fig. 2b). The majority (84%) of responsive units were observed in the superior temporal plane and the superior temporal gyrus, but responsive units were also detected in other lateral temporal sites, such as the middle temporal gyrus, and in the orbitofrontal cortex (Fig. 2c and Extended Data Fig. 3). Responses recorded in the posteromedial Heschl’s gyrus, probably corresponding functionally to A1 (refs. ^33,36^) (*n* = 236 responses in 33 clusters), were not significantly attenuated in NREM sleep compared with waking (gain = −15.25%, *P* = 0.30), whereas those in regions outside A1 (*n* = 91 responses in 22 clusters) showed a significant attenuation (gain = −40.09%, *P* = 0.001; see Supplementary Table 2 for direct comparisons between responses in A1 and non-A1 regions). Despite the overall pattern of attenuation in response magnitudes, robust high-fidelity responses persisted during NREM sleep also in regions outside A1, as exemplified by the activity of a non-A1 neuronal cluster in response to presentation of an excerpt of Mozart music (Supplementary Video 1). Indeed, mutual information (MI) between the auditory stimulus and the spiking response was only moderately attenuated during NREM sleep compared with wakefulness, (gain = −17.0%, *P* = 0.033). Separate analysis per region did not reveal significant MI attenuation in A1 units (gain = −7%, *P* = 0.44), but only outside A1 (gain = −31%, *P* = 0.01; Supplementary Table 2). Thus, robust and selective auditory spiking responses across the temporal lobe persist during NREM sleep and show only moderate attenuation in response magnitude.

**Fig. 3:**
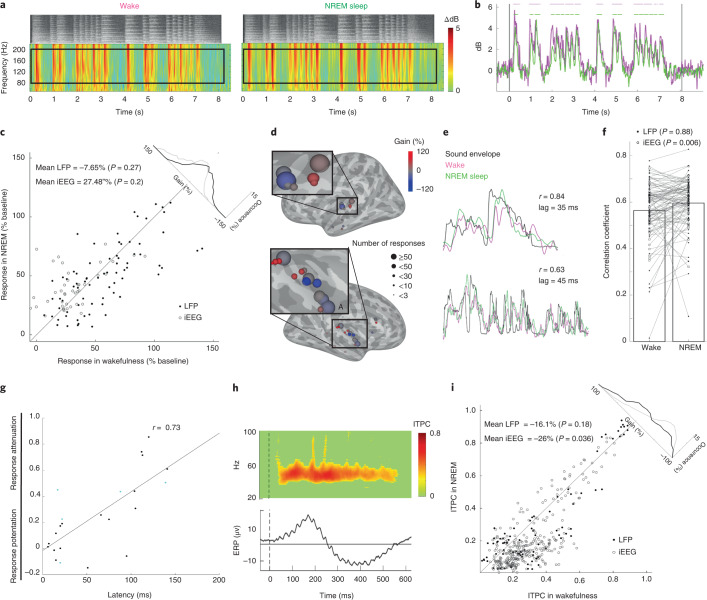
High-gamma auditory responses and entrainment to 40-Hz click-trains during NREM sleep. **a**, Representative spectrogram of induced LFP high-frequency power in response to music during wakefulness (left) and NREM sleep (right). Color bar on right. Black rectangles represent time–frequency regions-of-interest used for subsequent quantification. Top, grayscale soundwave spectrograms (lighter shades denote stronger power). **b**, Time-courses of high-gamma (80–200 Hz) responses shown in **a**. Horizontal bars and vertical black lines as in Fig. 2a. **c**, Scatter plot of high-gamma responses (% increase from baseline) during NREM sleep (*y* axis) versus wakefulness (*x* axis). Gain histogram in upper-right inset as in Fig. 2b; black and gray lines represent distributions for LFP and iEEG data, respectively. Each data point represents the averaged response across stimuli and trials per electrode (*n* = 556 responses/74 LFP microwires, black dots (CI (−23.732, 8.426), *P* = 0.276); 320 responses/55 iEEG channels, white circles (CI (−0.461, 55.422), *P* = 0.205); six patients). **d**, High-gamma gain values (NREM versus wakefulness) in each region exhibiting auditory high-gamma responses. Circle positions, color and size as in Fig. 2c. The letter A marks location of the representative microwire shown in panel **a** and **b**. **e**, Representative time-courses of LFP high-gamma responses showing a tight relationship with the sound envelope of auditory stimulus. **f**, Robust correlation between LFP high-gamma responses and the sound envelope in both wakefulness and NREM sleep. LFP: *r*(NREM-wake) = −0.002, CI (−0.02, 0.03), *P* = 0.88 for *n* = 406 responses/64 microwires/6 patients; iEEG: *r*(NREM-wake) = 0.04, CI (−0.07, −0.01), *P* = 0.006 for *n* = 210 responses/40 macroelectrodes/6 patients. **g**, Scatter plot of the degree of response attenuation in NREM sleep (*y* axis) versus latency of gamma LFP response (*x* axis) in each microwire (*n* = 25); Pearson correlation coefficient: *r* = 0.73, *P* < 0.001 by permutation test. Cyan dots mark adjacent microwires that exhibit different sleep attenuations and latencies. **h**, iEEG ITPC in response to a 40-Hz click-train in wakefulness (top), and associated Event-Related Potential (ERP) (bottom). **i**, Scatter plot of ITPC in response to 40-Hz click-trains during NREM sleep (*y* axis) versus wakefulness (*x* axis). Inset and format as in panel **c**. Each data point in scatter represents the averaged response across stimuli and trials per electrode. *n* = 84 LFP microwires/12 patients (black dots, CI (−40.9, 8.7), *P* = 0.176) and *n* = 325 iEEG macroelectrodes/13 patients (white circles, CI (−49.9, −2.0), *P* = 0.036). Mean and *P* values were calculated using a nested mixed model analysis for panels **c** and **f** and a one-level mixed model for panel **i** (Methods). Source data

**Extended Data Fig. 4:**
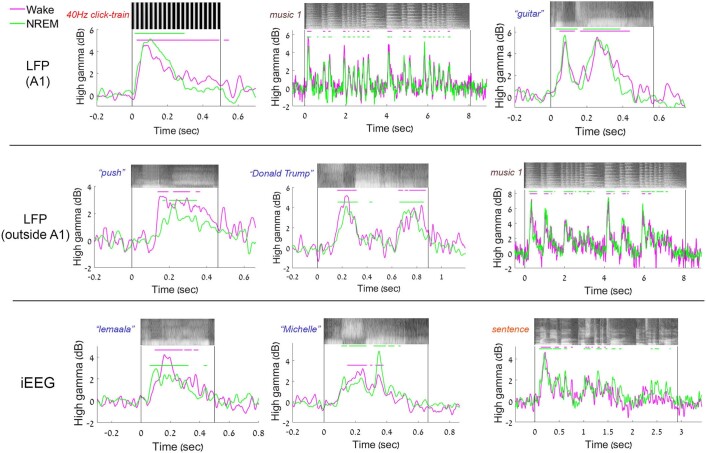
Additional examples of LFP and iEEG induced high-gamma auditory responses during wakefulness and NREM sleep. LFP and iEEG induced high-gamma (80–200 Hz) power time-courses during wakefulness (pink) and NREM sleep (green) in response to different type of stimuli (words in blue, sentence in orange, 40 Hz click-train in red, music in brown). Grayscale soundwave spectrograms are shown above each raster (lighter shades denote stronger power).

**Extended Data Fig. 5:**
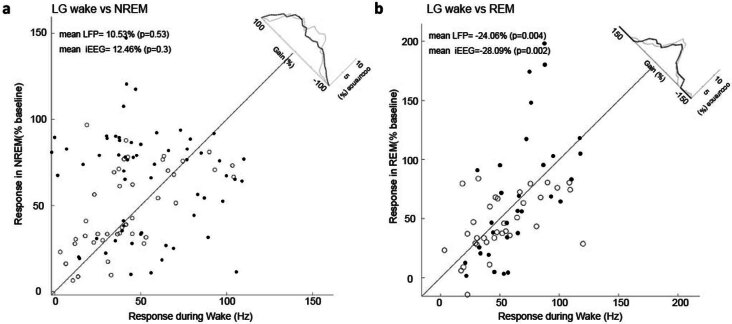
Low gamma responses during NREM and REM sleep compared to wakefulness. (a) Scatter plot of all low-gamma (40–80 Hz) averaged response magnitudes (% increase from baseline) per channel (mean gain = +10.53%, n = 417 responses/ 61 LFP microwires / five patients (black dot, p = 0.53, CI [-36.712, 57.781]) and mean gain: +12.46%, 293 responses/ 43 iEEG macrowires / seven patients (white circle, p = 0.3, CI [-19.986, 44.912]) in NREM sleep (y-axis) vs. wakefulness (x-axis), together with a histogram of gain values comparing response magnitude (upper-right corner along the unity diagonal, black and gray lines in top right inset represent gain distributions for LFP and iEEG data, respectively). (b) Same as (a) in REM sleep. (mean gain = −24.06%, p = 0.004, CI [-39.552, -8.562], n = 285 responses/ 31 LFP microwires/ two patients (black dot) and mean gain = -28.09%, p = 0.002, CI [-44.166, -12.022], 200 responses/ 34 iEEG channels / four patients (white circle)). Data point represent the averaged response per channel. Mean and p-value were calculated using a mixed model analysis (see Methods). In LFP microwires, neither anatomical location (A1 vs outside A1) or stimulus type affected the response magnitude during sleep (p = 0.27 and p = 0.49, respectively). Source data

**Extended Data Fig. 6:**
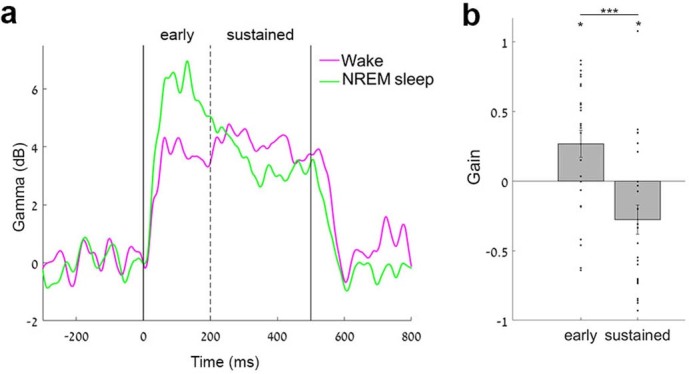
Factors associated with the degree of auditory response attenuation in NREM sleep. **(a)** Representative low-gamma response to a 40 Hz click-train in wakefulness (pink) and NREM sleep (green) shows differences between early vs. sustained response components. **(b)** Quantitative analysis across all low-gamma responses to 40 Hz click-trains (mean ± SEM across n = 25 LFP microwires) reveals that sustained responses show a stronger attenuation than early response during NREM sleep (p = 5.1*10^-05^ by two sided Wilcoxon signed-rank test). Early responses were actually slightly potentiated during NREM sleep (positive gain of 0.27 p = 0.016 via signed-rank test): ***p < 0.001. Source data

**Extended Data Fig. 7:**
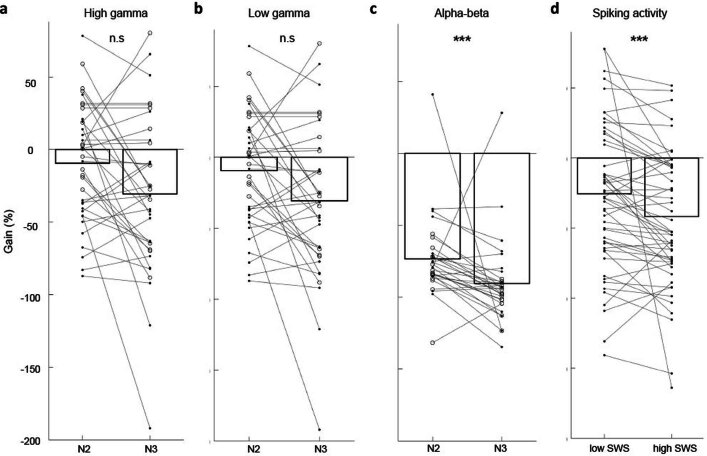
Responses during N3 and high slow waves activity show more attenuation. **(a)** High gamma gain during N3 vs N2 (black dots: n = 156 responses/ 22 LFP microwires / two patients, gain in N2 = −38.61 % vs in N3 = 41.78 %, p = 0.396; white circles: n = 53 responses/ 15 iEEG macroelectrodes / three patients, gain in N2 = −12.73% vs in N3 = −17.7%, p = 0.398). **(b)** Same for low gamma (black dots: n = 93 responses/ 15 LFP microwires / two patients, gain in N2 = −21.56% vs in N3 = 29.2%, p = 0.14; white dots: n = 123 responses/ 25 iEEG macroelectrodes / four patients, gain in N2 = −21.63% vs in N3 = −31.80%, p = 0.059). **(c)** Same for alpha-beta desynchronization (black dots: n = 105 responses/ 15 LFP microwires, gain in N2 = −73.48% vs in N3 = −84.00%, p < 0.001; white dots: n = 122 responses/ 16 iEEG macroelectrodes, gain in N2 = −86.03% vs in N3 = −98.72%, p < 0.001). The number of patients is lower because we only used sessions with full PSG (as automatic sleep scoring did not allow us to differentiate between N2 and N3) and that include N2 and N3 epochs. **(d)** Auditory spike responses during NREM sleep with high (top 20%) SWA show stronger attenuation compared to periods of low (bottom 20%) SWA (gain for low SW = −27% vs for high SW = −41%, p < 0.001). A similar effect (not shown graphically) was found for periods of high sigma (10–16 Hz) power representing spindle activities (gain = −19.5%, p = 0.001, n = 263 responses/55 clusters/six patients). Response magnitudes in spiking activity in deep vs. shallow NREM sleep were compared in high versus low SWA, rather than in N3 vs. N2 sleep (more simultaneous data allowing for paired comparisons). Data point represent the averaged response per channel. Mean and p-value were calculated using an one level (channels) mixed model analysis. Source data

Next, we focused on auditory-induced high-gamma (80–200 Hz) power responses, which are known to be closely linked to neuronal firing rates in human auditory cortex^37^, and compared them across wakefulness and NREM sleep. The results revealed highly robust auditory-induced high-gamma responses (Fig. 3a–d; additional examples in Extended Data Fig. 4). The magnitudes of high-gamma responses in NREM sleep were not significantly different from those in wakefulness (Fig. 3c,d; gain of −7.65%, *P* = 0.27 for LFP; gain of +27.48%, *P* = 0.2 for iEEG; see Supplementary Table 2 for responses in A1 and outside A1). The relationship of the high-gamma power envelope to the sound envelope was similar in LFPs across NREM sleep and wakefulness (Fig. 3e,f; *P* = 0.88, *r* = 0.58 in both wakefulness and NREM) and even slightly potentiated during sleep in iEEG data (*r* = 0.56 and 0.61 in wake and NREM, respectively, *P* = 0.006). Locking of high-gamma power to the sound envelope tended to be slightly higher in electrodes in A1 versus outside A1 (*P* = 0.097; Supplementary Table 2). We also analyzed low-gamma (40–80 Hz) responses and did not observe significant differences across wakefulness and NREM sleep (Extended Data Fig. 5 and Supplementary Table 2). The degree of high-gamma response attenuation during NREM sleep (gain) in each microwire was weakly correlated with the gain of spiking responses in neuronal clusters identified on that microwire (*n* = 221 responses/45 clusters/5 patients, *r* = 0.14, *P* = 0.038). The degree of response attenuation during sleep was strongly correlated with the response latency (Fig. 3g; *r* = 0.73, *P* < 0.001 by permutation test). In addition, late/sustained components of the auditory response (>200 ms) were more strongly reduced during sleep than early (<200 ms) response components (Extended Data Fig. 6). Other factors such as the degree of slow wave activity (SWA, power < 4 Hz), trials occurring in N3 versus N2 sleep, as well as sigma (10–16 Hz) power representing sleep spindle activities, were also associated with greater reduction of auditory response magnitudes during sleep (Extended Data Fig. 7). Comparing the degree of entrainment to fast stimulus modulations as described previously^33^, we found that 40-Hz click-trains in wakefulness strongly entrained field potentials (Fig. 3h). During NREM sleep (Fig. 3i), iEEG entrainment was attenuated by −26.0% (*P* = 0.036) whereas entrainment in LFPs did not show significant attenuation (−16.15%, *P* = 0.18) (see Supplementary Table 2 for A1 versus outside A1 comparison). Altogether, high-gamma responses in NREM sleep were robust and comparable to those in wakefulness apart from in some specific conditions (for example, high latency responses or deepest sleep), and entrainment to 40-Hz click-trains was moderately attenuated during NREM sleep compared with waking.

### Alpha–beta desynchronization (ABD) induced by auditory stimulation is disrupted during sleep

**Fig. 4:**
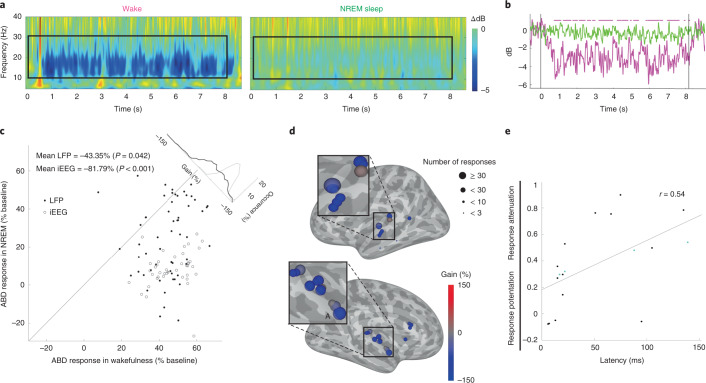
NREM sleep disrupts auditory-induced LFP ABD. **a**, Representative spectrogram of auditory-induced LFP power (<50 Hz) in response to music during wakefulness (left) and NREM sleep (right). Colder colors (for example, blue) denote a decrease in power (dB scale, color bar on right). Black rectangles represent time–frequency regions-of-interest used for subsequent quantification. **b**, Time-course of induced alpha–beta (10–30 Hz) power dynamics shown in **a**. Pink, wakefulness; green, NREM sleep. Horizontal pink bars above the time-course indicate automatically detected response intervals (Methods) for which the response magnitude was compared quantitatively (significant decreases were not detected in sleep). Vertical black lines mark stimulus onset and offset. **c**, Scatter plot of all auditory-induced ABD responses (% power decrease below baseline) during NREM sleep (*y* axis) versus wakefulness (*x* axis), together with a histogram of gain values comparing response magnitude (upper-right corner along the unity diagonal; black and gray lines in top-right inset represent distributions for LFP and iEEG data, respectively). Each data point in scatter represents the averaged response across stimuli and trials per electrode. *n* = 244 responses/57 LPF microwires/7 patients (black dots, CI (−84.434, −2.258), *P* = 0.042) and *n* = 188 responses/29 iEEG electrodes/5 patients (white dots, CI (−92.899, −70.678), *P* < 0.001). Mean and *P* values were calculated using a nested mixed model analysis. **d**, ABD gain values (NREM versus wakefulness) in each region exhibiting such responses. The position of each circle represents its anatomical location shown on a standard (MNI) brain template, the circle’s color reflects the average gain detected in that region (color bar on right) and the circle’s size reflects the number of responses detected in the region. The letter A marks the location of the representative microwire shown in panel **a**. **e**, Scatter plot of ABD gain values (*y* axis) versus latency of ABD (*x* axis) in each microwire (*n* = 18). Pearson correlation coefficient *r* = 0.54, *P* < 0.001 by permutation test. Cyan dots mark adjacent microwires that exhibit different sleep attenuations and latencies. Source data

**Extended Data Fig. 8:**
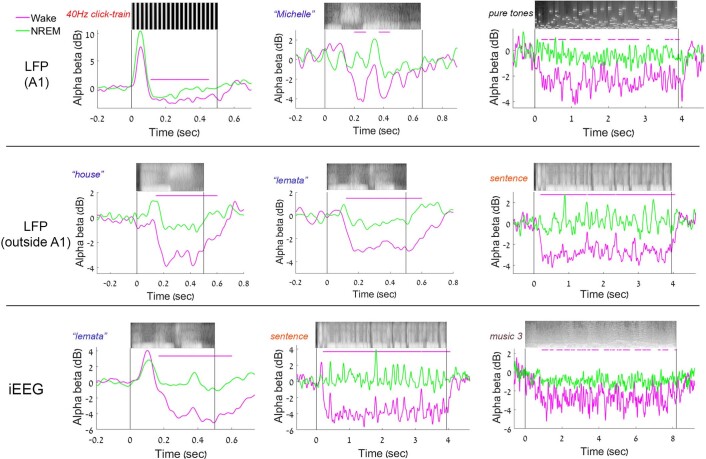
Additional examples of LFP alpha-beta auditory responses. LFP induced alpha-beta (10–30 Hz) power time-courses during wakefulness (pink) and NREM sleep (green) reveal disrupted alpha-beta responses during sleep in response to different type of stimuli (words in blue, sentence in orange, click-train in red, music in brown). Grayscale soundwave spectrograms are shown above each raster (lighter shades denote stronger power).

In humans, sensory responses often manifest as an increase in spiking activity and LFP high-gamma power, accompanied by a decrease in low-frequency power (also termed ‘desynchronization’)^37–41^. Accordingly, during wakefulness we observed strong auditory-induced ABD (10–30 Hz) (Fig. 4a,b and Extended Data Fig. 8). This auditory-induced ABD was strongly reduced during NREM sleep compared with waking (Fig. 4c,d, mean gain: −81.79 % and –43.35%, *P* < 0.001 and *P* = 0.042 for iEEG and LFP, respectively). Directly comparing ABD and high-gamma responses revealed that ABD responses were significantly more attenuated during sleep than high-gamma (37.61% and 113.71% greater attenuation for LFP and iEEG respectively, *P* < 0.001). ABD attenuation was modulated by stimulus type (*F*﻿_5,23.5_ = 5.3, *P* = 0.002 via linear mixed model), with least attenuation (most preserved responses) found for music (Supplementary Table 3). As observed for high-gamma responses, response latency correlated with ABD attenuation (Fig. 4e; *r* = 0.54, *P* < 0.001). Overall, NREM sleep robustly disrupts the ABD response to auditory stimuli.

### Auditory responses during rapid eye movement (REM) sleep

**Fig. 5:**
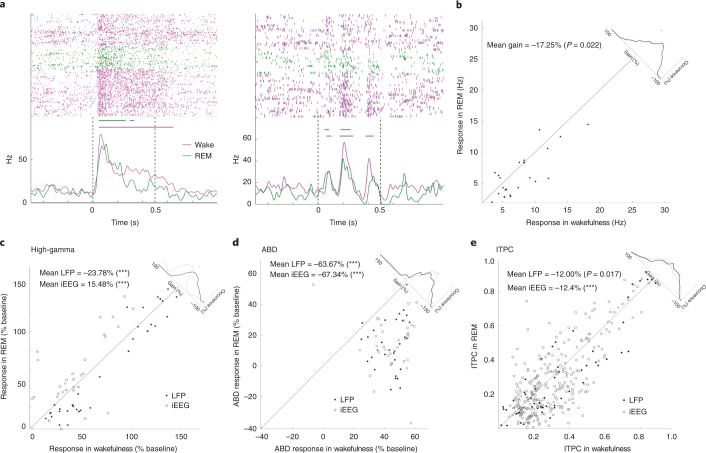
Auditory responses in REM sleep. **a**, Two representative raster plots (top) and PSTHs (bottom) of spiking response of neuronal units to auditory stimuli (left, click-train; right, word) in the primary auditory cortex. Pink, wakefulness; green, REM sleep. Vertical dotted black lines mark stimulus onset and offset. Horizontal bars above the PSTH time-courses indicate automatically detected response intervals (Methods) for which the magnitude of the response was compared quantitatively. **b**, Scatter plot of auditory spiking response magnitudes during REM sleep (*y* axis) versus wakefulness (*x* axis), together with a histogram of gain values comparing response magnitudes (upper-right corner along the diagonal). *n* = 141 responses/25 clusters/2 patients (CI (−31.763, −2.739), *P* = 0.022). **c**, Scatter plot of high-gamma responses to auditory stimuli during REM sleep (*y* axis) versus wakefulness (*x* axis), with a histogram of gain values comparing response magnitude (upper-right corner along the unity diagonal; black and gray lines in top-right inset represent gain distributions for LFP and iEEG data, respectively). Each data point represents the averaged response across stimuli and trials per electrode. *n* = 286 responses/33 LFP channels/2 patients (CI (−34.726, −12.838), *P* < 0.001) and *n* = 197 responses/30 iEEG channels/3 patients (CI (8.328, 22.630), *P* < 0.001)). **d**, Scatter plot of ABD responses to auditory stimuli in REM sleep (*y* axis) versus wakefulness (*x* axis). Histograms in top-right inset represent gain distributions above. *n* = 154 responses/32 LFP channels/3 patients (CI (−75.132, −52.207), *P* < 0.001) and *n* = 217 responses/36 iEEG channels/4 patients (CI (−78.814, −55.867), *P* < 0.001). **e**, Scatter plot of ITPC in response to 40-Hz click-trains during REM sleep (*y* axis) versus wakefulness (*x* axis), with a histogram of gain values as above. *n* = 60 LFP microwires/8 patients and *n* = 326 iEEG electrodes/9 patients. Each data point represents the averaged response across trials per electrode. Mean and *P* values were calculated using a one-level mixed model analysis (Methods); ***P < 0.001. Source data

**Extended Data Fig. 9:**
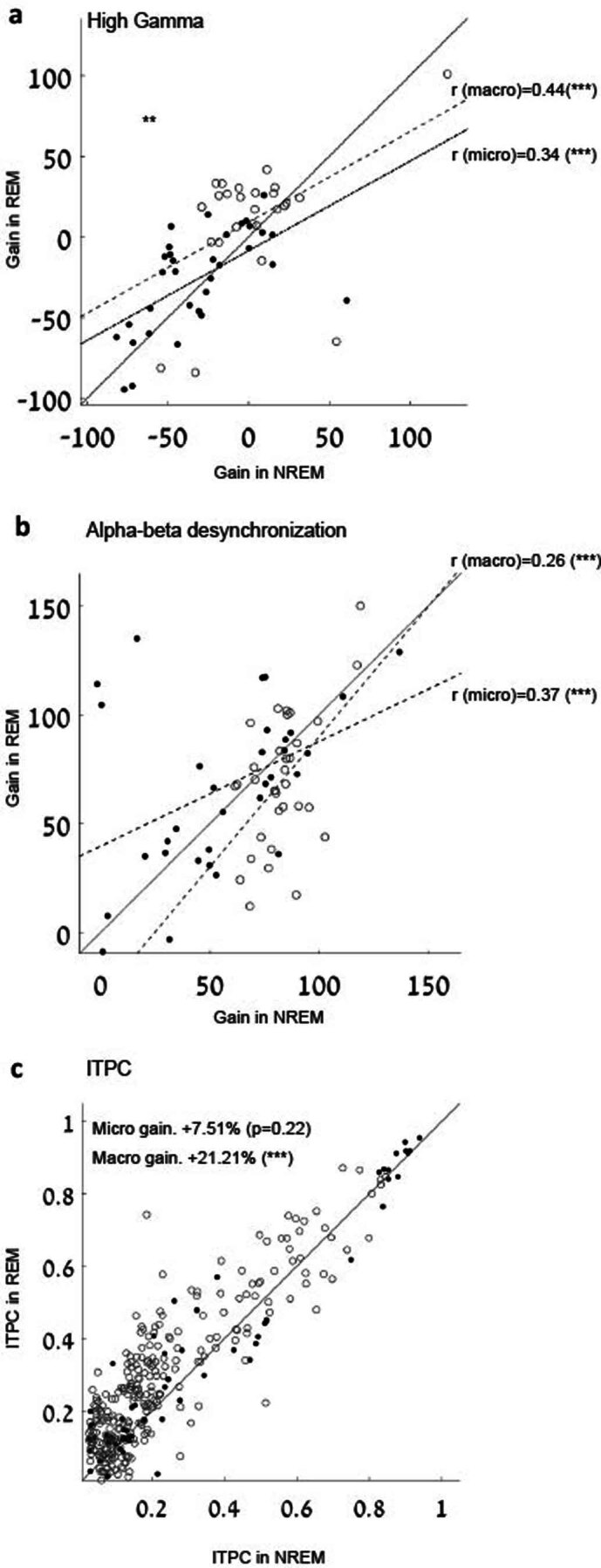
Correlation between gain during NREM and REM sleep. **(a)** High gamma power auditory responses (n = 32 LFP microwires (black dots) and 30 iEEG macroelectrodes (white circles) in REM sleep (y-axis) vs. NREM sleep (x-axis) show significant correlation. Each dot represents the averaged response per channel **(b)** Same as (a) for ABD (n = 32 LFP microwires (black dots) and 36 iEEG electrodes (white circles)). **(c)** Scatter plot of inter-trial phase coherence (ITPC) of 40 Hz LFP (black dots) and iEEG (white dots) responses (n = 60 and 326 respectively) in REM sleep (y-axis) vs. NREM sleep (x-axis); ***p < 0.001. Data point represent the averaged response per channel. Mean and p-value were calculated using a mixed model analysis. Source data

Lastly, we examined the auditory responses during REM sleep (Fig. 5a,b; *n* = 9 sessions in eight patients). Compared with wakefulness, response magnitude in spiking activity was moderately attenuated (gain of −17.25%, *P* = 0.022), and MI showed a trend for slight attenuation (gain = −12.1%, *P* = 0.065). MI was significantly more preserved in REM sleep than in NREM sleep (*P* = 0.002; Supplementary Table 4). The magnitude of induced high-gamma responses in REM sleep (Fig. 5c; see Extended Data Fig. 5b for low-gamma) was slightly attenuated in LFPs (gain = −23.78%, *P* < 0.001) but slightly potentiated in iEEGs (gain = 15.48%, *P* < 0.001). By contrast, ABD was robustly disrupted in REM sleep, as was the case during NREM sleep (Fig. 5d): gain = −63.67% for LFPs (*P* < 0.001) and gain = −67.34% for iEEG (*P* < 0.001). The correlation between high-gamma and the sound envelope was somewhat attenuated during REM sleep compared with waking (from 0.51 in awake to 0.44 in REM sleep for LFPs, *P* < 0.001; from 0.44 to 0.41 for iEEGs, *P* = 0.20). The degree of attenuation during REM sleep was correlated with the degree of attenuation during NREM sleep in the same electrode (*P* < 0.001; Extended Data Fig. 9a,b). As in NREM sleep, the attenuation of the ABD during REM sleep (versus waking) was significantly greater than the attenuation of the high-gamma response (35.55% and 74.49% greater attenuation, *P* < 0.001 for LFP and iEEG, respectively). Entrainment to 40-Hz click-trains during REM sleep was slightly attenuated compared with wakefulness (Fig. 5e; see Supplementary Table 4 for direct comparisons between NREM and REM sleep). Overall, most auditory responses during REM sleep were qualitatively similar to those observed during NREM sleep, but in some signals the response attenuation during REM sleep was more modest (responses were slightly more similar to wakefulness). Importantly, robust ABD attenuation co-existing with extensive spiking and high-gamma responses persisted also in REM sleep.

## Discussion

In summary, our results reveal robust neuronal and high-gamma auditory responses during sleep in Heschl’s gyrus and also in the anterior Superior Temporal Gyrus (STG), planum polare and middle temporal gyrus—well beyond early auditory cortex. Compared with responses during wakefulness, responses during sleep were either not significantly smaller or were only moderately smaller and any response attenuations were most pronounced for late sustained responses. Responses during sleep continued to track the envelope of auditory sound waves as they did in wakefulness, and their information content was only slightly reduced compared with that during wakefulness. Functionally, the 17% difference in MI represents a moderate change; for example, a recent study examining MI in the gerbil primary auditory cortex showed a threefold decrease between responses in desynchronized and synchronized anesthesia states^42^. In contrast to the robust spiking and high-gamma responses observed during sleep, auditory-induced ABD was significantly smaller during both NREM and REM sleep compared with waking. Finally, entrainment of field potentials to fast stimulus modulation rates (40-Hz click-trains) was reduced during NREM sleep compared with waking, but was more comparable between the desynchronized states of REM sleep and wakefulness. Our results establish that extensive and robust auditory responses persist during sleep while ABD (power decrease) is reduced.

Some limitations of the study should be explicitly acknowledged. First, we cannot entirely rule out the contribution of epileptiform activity. However, we carefully removed epochs including signs of interictal epileptic activity from the analysis and the highly consistent results observed across patients with different clinical profiles argue against a major contribution by pathology, and we do not believe this plays a major role or affects the conclusions. Second, the number of channels used in the latency versus gain analysis was relatively small, which is suboptimal for correlation analysis; however, the observation that response latency is correlated with response attenuation during sleep is also reported in a recent comprehensive rodent study with many more electrodes^20^. Third, the localization of the electrodes did not permit a distinction between cortical layers.

A unique aspect of the current study which we regard as a strength is that we used a passive auditory stimulation paradigm. While this approach may have limited the extent of responses, importantly it allowed us to address changes related to sleep per se, without the confound of post-perceptual processes (for example, related to report). Using a passive listening paradigm, we find that high-gamma activation was mainly restricted to electrodes located in the temporal lobe, contrary to several studies that showed a prominent activation of the prefrontal cortex associated with a P3 wave when auditory stimulation was associated with a task^27,43^. Instead, our results are in line with a recent EEG study showing little frontal involvement in auditory processing^44^. Thus, our findings provide important data to the study of neural correlates of conscious processing in the absence of report^45^.

Our results demonstrate the presence of robust neuronal and high-gamma power responses in the early auditory cortex, with similar response magnitudes in sleep and wakefulness. This is consistent with recent animal^20,22–24^ and noninvasive human^10,26,27,31^ studies. There was a stronger attenuation during sleep for late sustained responses (Fig. 3g) and in NREM sleep compared with REM sleep, as recently observed in the rat^20^. Downstream from A1, responses were moderately attenuated but overall we observed robust and extensive responses during sleep across the lateral temporal lobe. In addition, spiking and high-gamma exhibited high-fidelity responses as evidenced by MI analysis and tight locking to soundwave amplitude. Several lines of evidence suggest that the gamma power responses likely represent feedforward (‘bottom-up’) processing^46–49^. Gamma oscillations are initiated in cortical input layer 4 and propagate to other cortical layers^46^. In addition, they are more readily observed in supragranular layers where feedforward projections originate^46–49^, they propagate from primary sensory regions to downstream high-level regions^46^, and blocking NMDA receptors and feedback processing boosts gamma power^46^. We therefore interpret our results as representing a state-invariant ‘feedforward sweep’^50^ in cortical sensory pathways that is tightly linked to physical stimulus features, but cannot elicit sensory awareness on its own, as is the case in unconscious^43,51^ conditions such as anesthesia^33^.

Some aspects of the auditory response in REM sleep resembled those during NREM sleep, whereas other aspects were more similar to those in wakefulness (Fig. 5 and Extended Data Fig. 9), thus mirroring the general notion that REM sleep represents a ‘paradoxical’ hybrid of NREM sleep and wakefulness. For example, some LFP/iEEG induced power changes (particularly the marked reduction in ABD) were similar across sleep states and significantly different from wakefulness. Other aspects of auditory processing such as the magnitudes of spiking responses or time-locked entrainment to fast stimulus modulation rates (locking to 40-Hz click-trains) were more similar across wakefulness and REM sleep, as observed also in a recent rat study^52^. Notably, NREM sleep and REM sleep share certain physiological aspects (for example, low monoamine neuromodulation and low muscle tone^6^) and phenomenological aspects (for example, disconnection from the external environment^6^). Other physiological aspects of REM sleep more resemble those in wakefulness (for example, high cholinergic tone, peripheral autonomic activation^6^) and the states also share certain phenomenological aspects (for example, the ability to generate conscious experience). Accordingly, we find that auditory responses in REM sleep represent a hybrid of elements observed in wakefulness and NREM sleep. Successful entrainment to fast stimulus modulation rates, which is strongest in wakefulness and REM sleep, is probably supported by desynchronized cortical activity enabled by high cholinergic tone^53^, which may facilitate conscious experience, including dreams.

Our results point to ABD as the most notable difference in sensory processing between wakefulness and sleep. ABD is readily observed in scalp EEG and intracranially upon auditory stimulation during wakefulness, even during passive listening^41,54,55^, as well as in other brain regions and sensory modalities^39,56^. Our results indicate that auditory-induced ABD during wakefulness is significantly disrupted during sleep (Fig. 4), as has been observed in anesthetic loss of consciousness^33^. Under conditions examined to date, ABD exhibits high correlation with the degree of high-gamma (although ABD is more spatially widespread) and the two phenomena can be parsimoniously described as a change in the exponent *χ* (‘slope’) of the 1/*f*^*χ*^ component of the power spectrum^57^. However, we did not detect a significant correlation between the degrees to which sleep affected ABD and high-gamma responses in individual electrodes, in line with other auditory studies suggesting that the two phenomena may be largely independent^33,41^. A number of studies implicate ABD in neural feedback processing. In the macaque, gamma power propagates from V1 to V4, representing feedforward processing, whereas alpha oscillations propagate from V4 to V1, mediating feedback processing^46^. Moreover, alpha (8–12 Hz) and beta (13–30 Hz) oscillations are maximal in infragranular layers^46–48^ where feedback connections arise^49^. ABD has also been shown to mediate feedback processing during speech processing in human intracranial EEG^58,59^ and during visual stimulation^60–62^, and is associated with better discrimination performance in the sensorimotor network^63^ and with the extent of auditory percepts in an illusory auditory paradigm^64^. The precise source of neural sensory feedback signals remains elusive; they may arise from distant fronto-parietal regions, or thalamic and reticular thalamic circuits^65^. Alternatively, given that neuronal responses and ABD were predominantly observed in the temporal lobe, neuronal feedback may be generated locally in high-order sensory regions, or even in recurrent networks of early sensory cortex. Neuromodulatory systems are also likely to play a role, given their mediation of cortical desynchronization^53^ and sensory perception^66^ and their reduced activity in sleep^67^.

Thus, our study suggests that impaired neural feedback signaling is a key feature of sleep and of sensory disconnection, even in REM sleep (which supports rich conscious experiences). Indeed, increasing evidence suggests that sleep and anesthesia may involve disruption of feedback processes^68–71^. Anesthesia, and other unconscious states (for example, vegetative states^72^), may decouple signaling along apical dendrites of layer 5 pyramidal neurons, thereby suppressing the influence of feedback arriving at the distal dendrites^73^. In conclusion, our results point to disrupted neural feedback signaling as a main feature of sleep, and to dissociation of feedforward and feedback signaling as a general attribute of unconscious states and sensory disconnection.

## Methods

### Patients

Thirteen patients with drug-resistant epilepsy (five females) were implanted with Behnke-Fried depth electrodes (Ad-tech)^74^ as part of their clinical pre-surgical evaluation to identify seizure foci for potential surgical treatment. Electrode locations were based solely on clinical criteria. All patients provided written, informed consent to participate in the research study, under the approval of the Institutional Review Board at the Tel Aviv Sourasky Medical Center (TASMC, nine patients), or the Medical Institutional Review Board at University of California, Los Angeles (UCLA, four patients). In total, 14 sessions (6 naps/8 nights) were recorded.

### Auditory stimulation

Auditory stimuli were delivered intermittently using a bedside speaker during naps or full-night sessions, where each recording session included periods of both wakefulness and sleep. Auditory stimuli were presented in a pseudo-random order, with the sound intensity level adjusted at the start of each session to be comfortably audible but not too loud (~42–52 dB Sound Pressure Level (SPL)), so the patients could sleep comfortably. Stimuli included 40-Hz click-trains, tone sequences, words, sentences and music sequences (duration range: 0.5–9.4 s). Word stimuli were compiled in English at UCLA and in Hebrew at TASMC. The type and number of stimuli varied between sessions, depending primarily on session length (nap versus overnight experiments). Accordingly, overnight experiments included 2,043 ± 841 trials of 40-Hz click-trains, 1,013 ± 359 trials of tone sequences, 5,334 ± 2,111 trials of different words, 385 ± 181 trials of sentences and 1,092 ± 374 trials of music sequences. Shorter nap experiments included 423 ± 82 trials of 40-Hz click-trains, 225 ± 56 trials of tone sequences, 1,170 ± 524 trials of different words, 104 ± 25 trials of sentences and 204 ± 47 trials of music sequences. One patient listened only to words and click-train and another one did not have tone sequences.

### Sleep staging

Full polysomnography (PSG: scalp EEG, electrooculogram, electromyogram and video) was recorded in seven sessions (3 nights/4 naps). Epochs were scored as wakefulness (W), N1/unknown, N2, N3 and REM sleep according to established guidelines^34^. In three sessions (2 nights/1 nap), only the scalp EEG signal was recorded together with intracranial data. In these cases, sleep scoring was performed using the scalp EEG, confirmed by visualization of iEEG spectrograms and video recordings. Periods scored as N2 and N3 displayed high levels of SWA and sigma (sleep spindle) activity, whereas periods of wakefulness and REM sleep were associated with low levels of SWA. For four sessions (two nights and two naps), sleep scoring was based on iEEGs and video recordings. We calculated time–frequency dynamics of the iEEG (spectrograms) using a 30-s window (without overlap) spanning frequencies from 0 to 40 Hz and averaged the power in the delta band (0.5–4 Hz). Epochs with delta power higher than the 55th percentile were scored as NREM sleep, and those with delta power lower than the 20th percentile were scored as wakefulness/REM sleep and were further subdivided: epochs where the video showed that the patient was awake (eyes open, moving, sitting) were scored as wakefulness. Long periods (>3 min) occurring during the second part of the night, where the video indicated that the patient was likely to be asleep (closed eyes, no movements), were scored as REM sleep. To further validate sleep scoring based solely on iEEG, we compared our automatic sleep scoring with manual scoring in the overnight sessions with full PSG. The results indicated that 81.47 ± 12.36%, 88.33 ± 6.68% and 84.44 ± 6.51% (*n* = 4 nights) of the epochs scored by automatic scoring as awake, NREM sleep and REM sleep, respectively, agreed with the scoring labels obtained by full PSG. Furthermore, performing data analysis only on patients with full PSG confirmed that all main results (spiking activity, low-gamma, high-gamma and ABD) are highly similar to those obtained when using the entire dataset.

### Electrophysiology

Each depth electrode had eight platinum iEEG contacts along the shaft (referenced to the scalp), together with eight microwires protruding 3–5 mm from the distal tip, and a ninth low-impedance reference microwire^74^ that served as reference for each microwire electrode bundle. Data were recorded using either Blackrock (30-kHz sampling rate) or Neuralynx (40-kHz sampling rate) data acquisition systems.

### Spike sorting

Neuronal clusters were identified using the ‘waveclus’ software package^75^ as described previously^35,76^: extracellular recordings were high-pass filtered above 300 Hz and a threshold of 5 s.d. above the median noise level was computed. Detected events were clustered (or categorized as noise) using automatic superparamagnetic clustering of wavelet coefficients, followed by manual refinement based on the consistency of spike waveforms and inter-spike interval distributions.

### Detection of significant spiking responses

We identified neuronal auditory responses as described previously^20^. First, the response in each trial was smoothed by convolution with a Gaussian kernel (*σ* = 10 ms). Next, a one-tailed Wilcoxon–Mann–Whitney test was used to compare the results across trials. Each millisecond (within an interval corresponding to the stimulus duration and the 100 ms following it) was compared against baseline activity (we corrected for the multiple comparisons using false-discovery rate^77^ with base alpha of 0.01). A minimum of six trials per condition (wakefulness or sleep states) was required. Components shorter than 5 ms were excluded, and undetected intervals shorter than 2 ms that preceded and followed responses were categorized as misses and bridged with adjacent intervals. To further reduce the risk of false detections, the total length of the response for each stimulus had to be greater than 1.5% of the stimulus length. Responses were normalized by subtracting the pre-stimulus baseline (0–500 ms) activity in each state (baseline normalization).

### Mutual Information (MI) analysis

To estimate how informative the spiking response of each unit was with respect to the set of temporally dynamic stimuli (various words, click-trains, music segments and tones), we divided each stimulus into 50-ms bins and calculated the number of spikes per bin for each trial and stimulus (for example, a word of 450-ms duration was segmented to 9 consecutive bins). We then pooled together the bins of all stimuli and calculated the MI between the two discrete variables of spike count in each bin (*r*, response) and the bin identity (*s*, stimulus):

$$MI\left( {r;s} \right) = \mathop {\sum }\limits_r \mathop {\sum }\limits_s p\left( {r,s} \right) \times \log \left( {\frac{{p(r,s)}}{{p\left( r \right) \ast p(s)}}} \right)$$

p refers to the probability of a given spike count (p(r)), bin identity (p(s)) or their intersection (p(r,s)). When comparing the MI between different behavioral states, the number of trials for each stimulus was equalized across states. Qualitatively similar results were obtained for 20-, 50- and 100-ms bins, suggesting that the choice of a 50-ms bin size did not affect the results.

### LFP and iEEG power analysis

Signals from macro- and micro-electrodes were down-sampled to 1 kHz and band-pass filtered at 40–80 Hz, 80–200 Hz and 10–30 Hz for low-gamma, high-gamma and alpha–beta frequency bands, respectively. They were then Hilbert-transformed to obtain the instantaneous amplitude envelope, and log converted to express their amplitude in dB. For each channel and frequency band, the baseline power was extracted from a 500-ms interval before trial onset, and the mean baseline power was subtracted from the response power, separately for each frequency band of interest and separately for each channel. Trials with power higher than 5 s.d. from the mean were excluded.

Time intervals associated with significant induced LFP power in response to auditory stimuli were detected with the same method described above for the neuronal spiking response. For LFP responses, response components shorter than 10 ms for low- and high-gamma (and 50 ms for alpha–beta) were excluded, and undetected intervals shorter than 4 ms that preceded and followed responses were categorized as misses and bridged with adjacent intervals. All responses were also inspected visually to rule out false automatic detections. These parameters were optimized after extensive visual inspection of automatic response detections; importantly, none of the results reported were dependent on the precise parameters used for response detection.

For latency analysis, the same automatic algorithm was applied on low-gamma filtered channels that exhibited a significant response to 40-Hz click-trains during the first 200 ms of the response interval. The first time point in this interval that showed significantly higher activity than baseline was defined as the response latency.

### Comparison across vigilance states (LFP analysis and spiking activity)

For each stimulus and pair of states to be compared (for example, wakefulness versus NREM sleep), we separately identified temporal intervals with significant responses in either state as described previously^20^.

We quantified the relation between response magnitudes in wakefulness and sleep using a gain factor as described previously^20,23,24,33^, after normalizing each response to the baseline of that state:

${\mathrm{Gain}} = \frac{{R_{{\mathrm{sleep}}} - R_{{\mathrm{awake}}}}}{{\max \left( {\left| {R_{{\mathrm{sleep}}}} \right|,\left| {R_{{\mathrm{awake}}}} \right|} \right)}} \times 100$, where *R*_sleep_ and *R*_awake_ are the response amplitudes for a specific cluster/channel during wakefulness or sleep.

### Analysis of correlation with soundwave envelope

LFP and iEEG channels with gamma band power modulations that displayed a significant response were further analyzed to quantify their correlation with the soundwave envelope (intensity dynamics). The soundwave envelope was extracted by calculating the running average of the square amplitude using a 5-ms window (without overlap). The high-gamma response was down-sampled to 200 Hz. We first identified, using cross-correlation, the temporal lag associated with the highest correlation. This was followed by calculation of the Pearson correlation between the response time-course and the soundwave envelope at this time lag, and analysis of the statistical significance using permutations (*P* < 0.01).

### Inter-trial phase coherence (ITPC) analysis of responses to 40-Hz click-trains

Responses to 40-Hz click-train were quantified using ITPC, calculated as described previously^33^. Briefly, ITPC was defined as: ${\mathrm{ITPC}} = \left| {\frac{1}{N}\mathop {\sum }\limits_{k = 1}^N {\mathrm{e}}^{{\mathrm{i}}\phi _k}} \right|$, where *N* represents the number of trials and *ϕ*_*k*_ the phase of the spectral estimate for trial *k* for the 40-Hz frequency.

### SWA

For each session, we calculated the power spectrum of the scalp EEG (or iEEG) in the 2-s interval preceding stimulus onset and extracted the SWA (0.5–4 Hz) and the sigma power (10–16 Hz). For each stimulus eliciting a significant response, we sorted the trials according SWA and separated trials occurring during low SWA (below the 20th percentile) or during high SWA (above the 80th percentile). A minimum of six trials in each category was required to include a specific channel in this analysis. We then compared the response for each stimulus between the two groups by Mann–Whitney tests.

### Statistics and mixed model analysis

No statistical methods were used to pre-determine sample sizes but our sample sizes are similar to those reported in previous publications^33,78,79^. Data distributions were assumed to be normal, but this was not formally tested. Data collection and analysis were not performed blind to the conditions of the experiments.

We used a nested (hierarchical) mixed linear model analysis with follow-up contrast throughout the manuscript with two levels (channels/clusters, and patients), unless stated otherwise. Corrections were only done when there were pairwise contrasts (when there were more than two means to compare), and there we used Tukey’s method.

Generally, mean values in scatter plots (for example, Figs. 2b, 3c,i, 4c and 5b–e) represent the mean of the responses to the different stimuli in each channel/unit, whereas estimates of mean effects in the Results section are those based on the linear mixed effects model, which may differ slightly due to differential contribution of patients in the number of channels/units contributed. Whenever the number of patients available for a specific analysis was less than five (for example, specific analyses for REM sleep) or when only one type of stimulus was used (for example, ITPC), we used a one-level mixed model (for channel/clusters or patient, respectively).

We fit a linear mixed model with a maximal random effect structure^80^. Analyses were carried out in R^81^ using the lme4 package^82^. All degrees of freedom were estimated using the Satterthwaite approximation^83^. When estimating Spearman (rank) correlations, we accounted for the hierarchical nature of the data by group-mean-centering the data. The statistical tests were performed two-sided, unless stated otherwise.

### Electrode localization

Pre-implant MRI scans (Siemens Prisma scanner or Magnetom Skyra or GE SIGNA scanner, 3T, T1 sequence, resolution 1 × 1 × 1 mm^3^ or 1 × 1 ×5 mm^3^) were co-registered with post-implant computed tomography scans (Philips MX8000 or Brilliance or Siemens Sensation-64, resolution 1.5 × 0.5 × 0.5 mm^3^ or 0.75 × 0.5 × 0.5 mm^3^) to identify the locations of the electrodes. Individual subject data were further transformed into brain average space to facilitate the simultaneous visualization of electrode positions in different individuals. Co-registration and localization were estimated by using FreeSurfer^84^ and BioImage^85^ software, according to the guidelines of iELVis^86^.

### Reporting summary

Further information on research design is available in the Nature Research Reporting Summary linked to this article.

## Online content

Any methods, additional references, Nature Research reporting summaries, source data, extended data, supplementary information, acknowledgements, peer review information; details of author contributions and competing interests; and statements of data and code availability are available at 10.1038/s41593-022-01107-4.

## Supplementary information

Reporting Summary

Supplementary Video 1 Neuron’s response to Mozart music during NREM sleep.

Supplementary Tables Supplementary Tables 1–4 and the datasets used for the different analyses with the output of the statistical tests performed.

## Source data

Source Data Fig. 2 Statistical source data.

Source Data Fig. 3 Statistical source data.

Source Data Fig. 4 Statistical source data.

Source Data Fig. 5 Statistical source data.

Source Data Extended Data Fig. 5 Statistical source data.

Source Data Extended Data Fig. 6 Statistical source data.

Source Data Extended Data Fig. 7 Statistical source data.

Source Data Extended Data Fig. 9 Statistical source data.

## Data Availability

Datasets supporting the findings of this paper are available in a Supplementary information excel file. Source data are provided with this paper.

## Code availability

Code supporting the findings of this paper are available on request from the corresponding authors.
