# Author Correction: Reduced neural feedback signaling despite robust neuron and gamma auditory responses during human sleep

**DOI:** 10.1038/s41593-022-01137-y

**Published:** 2022-07-19

**Authors:** Hanna Hayat, Amit Marmelshtein, Aaron J. Krom, Yaniv Sela, Ariel Tankus, Ido Strauss, Firas Fahoum, Itzhak Fried, Yuval Nir

**Affiliations:** 1 Department of Physiology and Pharmacology, Sackler School of Medicine, Tel Aviv University, Tel Aviv, Israel; 2 Sagol School of Neuroscience, Tel Aviv University, Tel Aviv, Israel; 3 Department of Anesthesiology and Critical Care Medicine, Hadassah-Hebrew University Medical Center, Faculty of Medicine, Hebrew University of Jerusalem, Jerusalem, Israel; 4 Functional Neurosurgery Unit, Tel Aviv Sourasky Medical Center, Tel Aviv, Israel; 5 Sackler Faculty of Medicine, Tel Aviv University, Tel Aviv, Israel; 6 EEG and Epilepsy Unit, Department of Neurology, Tel Aviv Sourasky Medical Center, Tel Aviv, Israel; 7 Department of Neurosurgery, University of California Los Angeles, Los Angeles, CA USA; 8 Department of Biomedical Engineering, Faculty of Engineering, Tel Aviv University, Tel Aviv, Israel; 9 The Sieratzki-Sagol Center for Sleep Medicine, Tel-Aviv Sourasky Medical Center, Tel-Aviv, Israel

**Keywords:** Sleep, Sensory processing

*Nature Neurology* 10.1038/s41593-022-01107-4, published online 11 July 2022.

In the version of this article initially published, “−2” and “−4” on the *y* axis of Fig. 5d should have been “−20” and “−40”. Further, the scale values near the spike waveforms in Fig. 2a, now reading “100 μs” appeared originally as “10 μs.” The errors have been corrected in the HTML and PDF versions of the article.

