# Supplementary material for: Reduced neural feedback signaling despite robust neuron and gamma auditory responses during human sleep
Source: Nat Neurosci. 2022 Jul 11;25(7):935–43. doi: 10.1038/s41593-022-01107-4 (PMC9276533; doi:10.1038/s41593-022-01107-4)
Supplement: Supplementary file 1 — Reporting Summary [file 41593_2022_1107_MOESM1_ESM.pdf]

nature portfolio

Corresponding author(s): Yuval Nir

Last updated by author(s): Apr 25, 2022

Reporting Summary

Nature Portfolio wishes to improve the reproducibility of the work that we publish. This form provides structure for consistency and transparency in reporting. For further information on Nature Portfolio policies, see our Editorial Policies and the Editorial Policy Checklist.

Statistics

For all statistical analyses, confirm that the following items are present in the figure legend, table legend, main text, or Methods section.

n/a	Confirmed	
☐	☒	The exact sample size (*n*) for each experimental group/condition, given as a discrete number and unit of measurement
☐	☒	A statement on whether measurements were taken from distinct samples or whether the same sample was measured repeatedly
☐	☒	The statistical test(s) used AND whether they are one- or two-sided<br>*Only common tests should be described solely by name; describe more complex techniques in the Methods section.*
☐	☒	A description of all covariates tested
☐	☒	A description of any assumptions or corrections, such as tests of normality and adjustment for multiple comparisons
☐	☒	A full description of the statistical parameters including central tendency (e.g. means) or other basic estimates (e.g. regression coefficient) AND variation (e.g. standard deviation) or associated estimates of uncertainty (e.g. confidence intervals)
☐	☒	For null hypothesis testing, the test statistic (e.g. *F*, *t*, *r*) with confidence intervals, effect sizes, degrees of freedom and *P* value noted<br>*Give P values as exact values whenever suitable.*
☒	☐	For Bayesian analysis, information on the choice of priors and Markov chain Monte Carlo settings
☐	☒	For hierarchical and complex designs, identification of the appropriate level for tests and full reporting of outcomes
☐	☒	Estimates of effect sizes (e.g. Cohen's *d*, Pearson's *r*), indicating how they were calculated

Our web collection on statistics for biologists contains articles on many of the points above.

Software and code

Policy information about availability of computer code

Data collection	Data from neurosurgical patients implanted with depth electrodes were collected during sleep and wakefulness while intermittently presenting auditory stimuli. Code used to present auditory stimuli is available on request from the corresponding authors
Data analysis	Data analysis was performed in Matlab 2017 using custom-developed analysis routines. Electrode localization was performed using iELVIS (based on FreeSurfer v6 and BioImage (legacy version) software). Data analysis code is available on request from the corresponding authors.

For manuscripts utilizing custom algorithms or software that are central to the research but not yet described in published literature, software must be made available to editors and reviewers. We strongly encourage code deposition in a community repository (e.g. GitHub). See the Nature Portfolio guidelines for submitting code & software for further information.

Data

Policy information about availability of data

All manuscripts must include a data availability statement. This statement should provide the following information, where applicable:

- Accession codes, unique identifiers, or web links for publicly available datasets
- A description of any restrictions on data availability
- For clinical datasets or third party data, please ensure that the statement adheres to our policy

Data sets supporting the findings of this paper are available in a supplementary information excel file.

March 2021

Field-specific reporting

Please select the one below that is the best fit for your research. If you are not sure, read the appropriate sections before making your selection.

☒ Life sciences ☐ Behavioural & social sciences ☐ Ecological, evolutionary & environmental sciences

For a reference copy of the document with all sections, see nature.com/documents/nr-reporting-summary-flat.pdf

Life sciences study design

All studies must disclose on these points even when the disclosure is negative.

Sample size	Rare data from neurosurgical patients participating in research during sleep were collected over 7 years. We recorded intracranial EEG (iEEG, n = 987 contacts), LFPs (n =937 microwires), and neuronal spiking activity (n = 713 clusters) from multiple cortical regions in 13 drug-resistant epilepsy patients implanted with depth electrodes for clinical monitoring (14 sessions). At least one depth electrode in each monitored individual targeted auditory (or other lateral temporal) cortical regions (a limited subset of all monitored patients).<br>No sample size calculation was performed, but our sample sizes are similar to those reported in previous publications. We are confident that the sample size is sufficient since the main findings are highly significant statistically, and can be observed in data of individual participants.
Data exclusions	No data exclusion
Replication	The experiment across sleep and wakefulness was repeated across 14 sessions (8 full-night sessions and 6 daytime nap session). The main findings were reliably reproduced across individual channels /sessions (using a linear nested mixed model analysis)
Randomization	Randomization was not applicable to the study since the main conditions are endogenous vigilance states (wakefulness, NREM sleep, REM sleep) that arise spontaneously, unrelated to the experimental design.
Blinding	Blinding was not applicable to the study, since whether subjects were awake or asleep is apparent in their electrophysiological and behavioral data.

Reporting for specific materials, systems and methods

We require information from authors about some types of materials, experimental systems and methods used in many studies. Here, indicate whether each material, system or method listed is relevant to your study. If you are not sure if a list item applies to your research, read the appropriate section before selecting a response.

Materials & experimental systems

n/a	Involved in the study	
☒	☐	Antibodies
☒	☐	Eukaryotic cell lines
☒	☐	Palaeontology and archaeology
☒	☐	Animals and other organisms
☐	☒	Human research participants
☒	☐	Clinical data
☒	☐	Dual use research of concern

Methods

n/a	Involved in the study	
☒	☐	ChIP-seq
☒	☐	Flow cytometry
☒	☐	MRI-based neuroimaging

Human research participants

Policy information about studies involving human research participants

Population characteristics	Neurosurgical epileptic patients implanted with depth electrodes for monitoring (8 males, 5 females , mean age = 32.1, age range: 17-43). Additional details on patients can be found in Supplementary Table 1.
Recruitment	Participants were recruited by the epilepsy neurosurgery staff at Tel Aviv Sourasky Medical Center (TASMC, 9 patients), or at UCLA (4 patients). Every patient to be implanted depth electrodes for clinical monitoring was approached to check for possible participation in research. We are not aware of any biases that may be present and impact the results.
Ethics oversight	All patients provided written informed consent to participate in the research study, under the approval of the Institutional Review Board at the Tel Aviv Sourasky Medical Center (TASMC, 9 patients), or the Medical Institutional Review Board at UCLA (4 patients).

Note that full information on the approval of the study protocol must also be provided in the manuscript.
